# Recent Advances in the Surgical Management of Radiation-Induced Fractures following Soft Tissue Sarcomas

**DOI:** 10.3390/jcm13113126

**Published:** 2024-05-27

**Authors:** Matteo Salvini, Alessandro El Motassime, Francesco Cavola, Pasquale Ruberto, Antonio Ziranu, Giulio Maccauro

**Affiliations:** 1 Orthopedics & Traumatology Unit, Fondazione Policlinico Universitario Agostino Gemelli IRCSS, 00168 Roma, Italy; 2 Orthopedics and Traumatology, Università Cattolica Del Sacro Cuore, 00168 Roma, Italy; 3 Orthopedics and Traumatology, Ospedale Isola Tiberina—Gemelli Isola, 00186 Roma, Italy

**Keywords:** radiation therapy, insufficiency fractures, irradiated bone, post-attinic, soft tissue sarcomas

## Abstract

**Background:** Post-radiation fractures are a significant complication of cancer treatment, often being challenging to manage and impacting patients’ quality of life. This study systematically reviews the literature on fractures in irradiated bones, focusing on risk factors, treatment modalities, and prevention strategies. Factors increasing fracture risk include exposure to high doses of radiation of at least 50 Gy, female gender, menopausal age, and periosteal stripping. Additionally further risk factors are the size of the original tumor and osteoporosis. **Methods:** A search of PubMed yielded 541 articles, with 4 were ultimately included in the review. These retrospective studies focused on patients undergoing Combined Limb-Sparing Surgery and Radiation Therapy for soft tissue sarcoma. **Results:** Results show post-radiation fractures affect approximately 4% of patients, with the femur being the most frequently affected site. Intramedullary nailing emerges as the gold standard treatment, with prosthetic replacement or megaprostheses used in the metaepiphyseal region and as salvage procedures. Non-union and infection remain formidable complications. **Conclusions:** This study highlights the importance of prophylactic nailing in fracture prevention and the efficacy of free vascularized fibular flaps to achieve bone union during revision surgeries. Limited case availability and patient follow-up hinder comprehensive studies, impacting treatment outcomes.

## 1. Introduction

In modern times, radiation therapy, also known as radiotherapy, plays a crucial role in cancer treatment. It involves the use of high-energy radiation to target and destroy cancer cells. This treatment modality has evolved significantly over the years, becoming more precise and effective while minimizing damage to healthy tissue.

One of the most significant advancements in radiation therapy is the development of new techniques such as Intensity-Modulated Radiation Therapy, Image-Guided Radiation Therapy, and Stereotactic Body Radiation Therapy [1]. These techniques have made it possible to target tumors more accurately, while minimizing harm to surrounding healthy tissues. For instance, IMRT can adjust the intensity of radiation beams to fit the shape of the tumor, thus reducing side effects and improving treatment outcomes.

Another notable development is the integration of radiation therapy with other treatment modalities like surgery, chemotherapy, and immunotherapy. This multidisciplinary approach, known as multimodal therapy, has led to better outcomes for many cancer patients by combining the strengths of different treatments to maximize effectiveness [2].

Over the past few years, there has been increasing interest in proton therapy, which is a form of radiation therapy that employs protons instead of traditional photon beams. Proton therapy provides the possibility of more precise targeting of tumors and decreased radiation exposure to healthy tissues. However, it is currently more expensive and less commonly available compared to conventional radiation therapy [3].

Overall, the use of radiation therapy in modern medicine continues to evolve, with ongoing research focused on improving treatment outcomes, reducing side effects, and expanding access to this important component of cancer care [4].

Radiation therapy can have both direct and indirect effects on bone tissue. Direct effects occur when radiation damages the cells within the bone, leading to changes in bone structure and density. Indirect effects result from damage to blood vessels, which can impair blood flow to the bone and hinder its ability to repair and regenerate [5].

One common complication of radiation therapy involving bones is radiation-induced fractures, also known as post-radiation fractures. These fractures can occur months to years after treatment (4 months to 21 years) [2] and are often characterized by poor healing due to compromised bone quality. Factors such as the dose and duration of radiation, as well as the location of the treated area, can influence the risk of developing post-radiation fractures [6]. As is reported in several studies, the incidence of fractures in irradiated bone is reported as 8.2% to 45.2% [7] with a consolidation rate of 33% to 75% [8].

Management of post-radiation fractures typically involves conservative measures such as pain management, bracing, and physical therapy. In some cases, surgical intervention may be necessary to stabilize the fracture and promote healing. However, these fractures can be challenging to treat and may have a significant impact on the patient’s quality of life.

These fractures are often associated with compromised bone quality due to the effects of radiation on bone cells and blood vessels. Several factors increase the risk of developing a post-radiation fracture, including: radiation dose and duration, as a higher dose (>60 Gy) [9], and prolonged or repeated courses of radiation therapy may increase the risk of bone weakening and fractures; location of radiation, as bones located near the radiation field and among those spine, pelvis, or long bones, are at increased risk of fractures; pre-existing bone conditions, such as osteoporosis or osteopenia; smoking, which is associated with lower bone density and impaired bone healing; concurrent treatments, such as periosteal stripping, which can thin the cortex and damage the periosteal vascularity, increasing the risk of fracture and of local ischemia, or chemotherapy, which may potentiate the effects of radiation on bone and increase fracture risk; hormonal imbalances or treatments may contribute to bone fragility; unbalanced nutritional status; and age, which is usually associated with lower bone density, but according to Hashimoto et al. [10], the first step in treating elderly patients should be surgery alone as they found out radiation and chemotherapy gave no additional benefits.

Managing the risk of post-radiation fractures involves careful assessment of these factors during treatment planning and monitoring for signs of bone damage during follow-up care. Strategies to reduce fracture risk may include lifestyle modifications, such as smoking cessation and regular exercise, as well as pharmacological interventions to support bone health, such as bisphosphonate treatment and monoclonal antibodies such as romosozumab [11].

Our study aims to systematically review the available literature on fractures in irradiated bones and investigate new perspectives for treatment or prevention.

We present the following article in accordance with the Preferred Reporting Items for Systematic Reviews and Meta-Analyses (PRISMA) reporting checklist (available at https://jxym.amegroups.com/article/view/10.21037/jxym-22-19/rc, accessed on 10 March 2024).

## 2. Materials and Methods

The systematic review we conducted involved identifying publications present in the literature on skeletal fractures resulting from prior exposure to ionizing radiation. 

We focused our attention on pertinent systematic reviews, case series, and reports. 

The research commenced by formulating a structured question and determining keywords, primarily sourced from the PubMed database. 

We conducted a search on the PubMed database (www.ncbi.nlm.nih.gov/pubmed) using the string “RADIOTHERAPY” AND “FRACTURE”, over the period of time from 2019 to 2023. 

No language limitation was applied to the research.

The title of the journal, name of authors, or supporting institutions were not masked at any stage.

**Figure 1:**
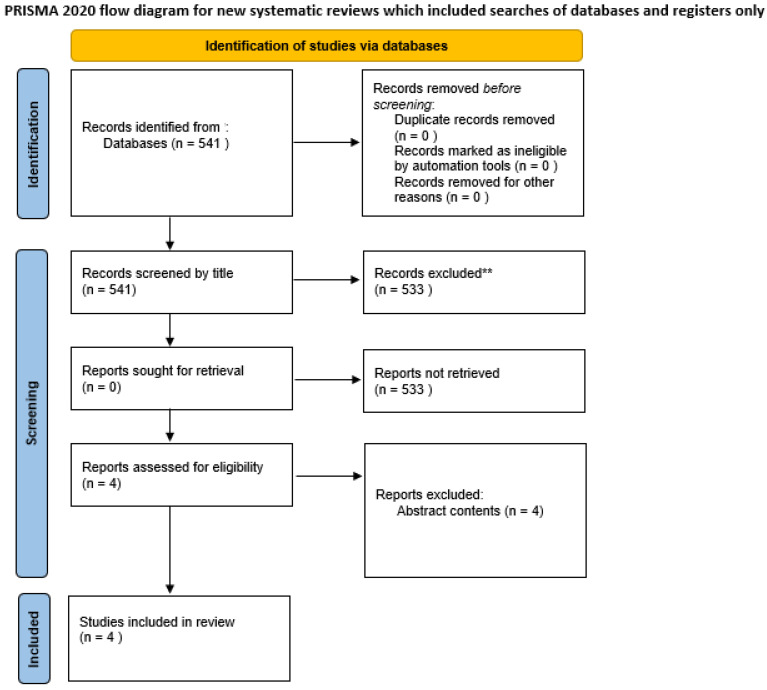
PRISMA flow chart.

This research, after applying the research criteria, yielded 541 articles. After the title and abstract analyses, we ended up with 8 articles. Of this group, 4 were excluded after the full text screening (Figure 1, PRISMA guidelines). 

The final group was gained by basing the screening on inclusion criteria that would focus the attention only on surgical treatment of primary fracture events, typically those that were spontaneous or due to low-energy trauma, not related to high-energy traumas, occurring after radiotherapy for soft tissue tumors such as sarcomas.

The results were screened by hand and we held back only the articles containing methods of treatment of the bone fractures.

Abstracts and full texts underwent separate examination by two authors (Cavola F., El Motassime A.), with any discrepancies resolved through agreement with a third author (Salvini M.). The methodological quality of the research was evaluated using the modified Coleman methodology score (mCMS). Two independent assessors evaluated each publication (Salvini M., El Motassime A.); in instances where there was a variance of more than five points in their assessment, a consensus was reached with a third author (Ruberto P.). The modified Coleman methodology score (mCMS) spans from 0 to 100 points, indicating a study free from bias or confounding variables (Table 1).

Claxton’s case analysis on the “utility of free vascularized fibular (FVF) flaps in post-radiation fracture non-unions in the upper extremity” is not directly relevant as it discusses a specific complication. However, they observed that the FVF flap technique is a reliable treatment option for radiation-associated non-unions of the upper extremities, achieving an overall union rate of 100% and an improvement in functional outcomes.

For the same reason, concerning the application of the FVF flap technique (vascularized fibular graft) in cases of femoral non-union, the article by Tibbo et al. [12] was excluded. The article specifically focuses on patients who experienced non-union complications after initial treatment, analyzing the outcomes of subsequent procedures (revision plate, exchange nail, etc.) only when performed in conjunction with the FVF flap technique. Following the second procedure, the reported union rate is 52%, increasing to 78% after a second operative grafting.

Once again, a complication is the topic in Matsuhashi’s paper [14] concerning the intricacies of postoperative infection in pathological femoral fractures following radiotherapy. The study presents two case reports and conducts a comprehensive review of the existing literature. The primary focus of their investigation lies in the application of the Masquelet technique for addressing necrotic bone and infected soft tissue at the surgical site.

The fourth article not included in the review was that of Razavian et al. [13] It is indeed a systematic review addressing “Radiation-Induced Insufficiency Fractures after Pelvic Irradiation for Gynecologic Malignancies” (PIFs). The issue lies in the fact that the article primarily aims to analyze the incidence, risk factors, clinical presentation of fractures, and characteristics of gynecologic tumors causing them, with a focus on the radiotherapy administered for their treatment. The surgical treatment aspect is briefly discussed as it is not gynecologically relevant and is applicable to only 9% of the cases analyzed. Approximately 85% of the fractures, mostly sacral, warranted conservative treatment (bed rest and analgesics), around 6% benefited from pharmacological treatment (bisphosphonates), and the remaining 9% underwent surgical interventions such as hip prosthetic replacement and vertebroplasty, with no analysis of the outcomes.

## 3. Results

From the final analysis of the full texts, we have thus included four articles in our review. These comprise four retrospective observational studies, two of which have a very limited patient series: three patients for the article by Bretschneider et al. [19] and seven patients for the article by Lee et al. [18] Additionally, two studies were included with larger patient cohorts of 16 and 28, in the articles by Sambri et al. [16] and Muratori et al. [17], respectively. However, the patient selection criteria were stringent across all articles. Specifically, these studies focused on patients who underwent Combined Limb-Sparing Surgery and Radiation Therapy (considered the gold standard) for soft tissue sarcoma, followed by post-radiation fractures (Table 2).

Only in Jongseok Lee et al.’s [18] paper, out of the seven patients included, two were not diagnosed with sarcomas. Specifically, one patient had melanoma, and another had a desmoid tumor. Furthermore, only in the study by Muratori et al. [17] were there eight patients with a sarcoma that did not originate in the thigh. Based on the studies examined, it can be highlighted that post-radiation fractures affect approximately 4% of patients undergoing radiotherapy in the context of soft tissue sarcoma treatment.

All patients included in the analysis received high-dose radiotherapy, at least >50 Gy, regardless of whether it was administered preoperatively, postoperatively, or both.

We can include 54 post-radiation fractures in our review. Among the four articles, Sambri et al.’s [16] is the only one addressing indications for prophylactic intramedullary nailing (PIN). Out of 16 fractures, only one patient underwent PIN, while the remaining 15 fractures reported in Table 2 did not receive this type of treatment. Additionally, Sambri et al. [16] noted that among the 16 patients who underwent periosteal stripping, half of them underwent PIN. Out of the total 11 patients who received PIN, only one fracture was reported (Table 3).

Approximately 67% of the patients were female. About 56% of the patients had undergone periosteal stripping. The study by Sambri et al. [16] is the only one that employs prophylactic intramedullary nailing (PIN), thus significantly reducing the percentage of patients who develop fractures following stripping; otherwise, the rates would be much higher. If we temporarily exclude the study by Sambri et al. [16], where half of the patients with stripping had undergone PIN, the percentage increases to 74%.

The average age at the time of the onset of insufficiency fractures (PIF—post-radiation insufficiency fracture) was between 55 and 60 years, and the mean time between radiotherapy and the onset of the fracture was 68 months (3–182). The femur is the most frequently affected site by post-attinic fractures, representing approximately 90% of fractures (Table 4).

The gold standard for post-radiation fractures of the long bones, particularly the femur and tibia, appears to be intramedullary nailing. In fact, in our investigation, this represents more than 60% of the performed surgical treatments. In the second position among primary treatments, we find prosthetic replacement or megaprostheses, especially in the metaepiphyseal region. These become the most commonly used treatment in surgical “salvage” procedures or at the conclusion of many osteosynthesis revisions for non-union. The PIN is a minimally utilized but promising approach, particularly effective in cases involving periosteal stripping or tangential resections. Non-unions are the most formidable complication, occurring in 60% of treated cases, which reduces to 45% after revision of osteosynthesis: thicker nail, dynamization, and blocking screws insertion when initial treatment was an intramedullary nail, whereas a longer plate was used with additional iliac crest bone grafting in the case of primary treatment with a plate. Plate osteosynthesis in the case of secondary treatments has consistently been accompanied by a bone graft. Only in the study by Muratori et al. [17] did we observe the use of Free Vascularized Fibular (FVF) grafts and autogenous iliac crest graft augmentation as a third attempt at revision with achievement of bone solidity.

## 4. Discussion

Radiotherapy has multiple effects on bone, ranging from decreased cortical bone strength to the inhibition of endochondral ossification and subsequent bone growth and healing. Radiotherapy is often utilized to assist with margin control in the setting of primary sarcoma and for palliative treatment in the setting of metastatic disease [15]. Specifically, irradiated bone is more susceptible to fracture because radiation damages the bone matrix by reducing the number of osteoblasts, increasing marrow adiposity, and degrading vascular supply [13]. 

Several risk factors for post-radiation fractures are reported in the literature. Certainly, through our study, we can confirm that female gender, exposure to high doses of radiation of at least 50 Gy, age between the fifth and sixth decades of life, and periosteal stripping are significant risk factors. Additionally, several articles report further risk factors such as the size of the original tumor, osteoporosis, and, notably, menopausal age. In addition to the aforementioned causes, known risk factors for insufficiency fractures include rheumatoid arthritis, corticosteroid use, autoimmune diseases, and metabolic bone diseases [13].

A risk factor not carefully analyzed in any of the studies included in the review is the grade of sarcoma excised. Considering that this influences patient prognosis, it is also possible that it affects the likelihood of post-radiation fracture [10].

Periosteal stripping has been confirmed as an additional risk factor. The periosteum supplies blood to the outer one-third of the bone cortex and possesses significant compensatory potential when vascularization is hindered by radiation-induced fibrosis. The combined effect of periosteal stripping and circumferential radiotherapy diminishes the blood supply to the bone segment. Consequently, osteonecrosis hinders the healing of fractures due to inadequate osteoblastic activity.

The statement emphasizes the challenges associated with managing PIF once they occur, highlighting the significance of prioritizing fracture prevention. It suggests the use of femoral prophylactic intramedullary nailing (PIN) in higher-risk patients as an intervention to reduce the likelihood of fractures. After periosteum stripping, it would be advisable to perform a PIN to reduce, though not entirely eliminate, the risks of post-radiation fracture.

Similarly, based on the issue of compromised vascularity, intramedullary nailing finds its rationale. It indeed prevents the wastage of the fracture hematoma and prevents, to the extent possible, further damage being caused to the fracture site. For this reason, it is the preferred treatment, where feasible. 

Nevertheless, non-union remains a highly probable and formidable complication. As our study indicates, it occurs at a rate of around 60%. In such cases, various revision procedures can be considered, such as using a thicker nail, dynamic fixation, or a complete revision of the osteosynthesis, where promising results seem to come from vascularized fibular grafts. It is important to consider that such procedures can only be performed in relatively young patients with adequate soft tissue conditions. In second or third interventions, infection becomes the most dreaded complication, often leading to unfavorable outcomes that result in limb amputation (18%).

**Figure 2:**
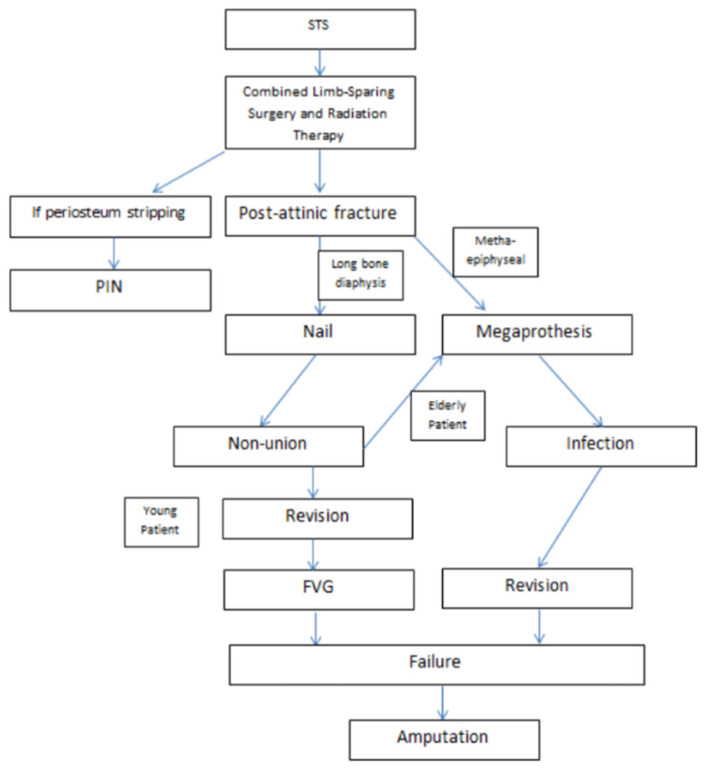
Flowchart Post-radiation Fractures.

Prostheses or megaprostheses seem to have excellent success rates when used as a primary treatment, whereas when employed as salvage therapy, they exhibit a significantly high complication rate, particularly in terms of infection. If we consider the two studies where prostheses are also applied as a primary treatment, there is an infection rate of 17%. However, only the study by Muratori et al. [17] provides data on the complication rate of megaprostheses used as salvage procedures, reporting an infection rate of approximately 30% (Figure 2).

## 5. Conclusions

The importance of a multidisciplinary approach in managing radiation-induced fractures, involving oncologists, radiologists, orthopedic surgeons, and rehabilitation specialists to optimize patient outcomes, has been widely demonstrated since the last century [20]. With the refinement of techniques, it becomes increasingly relevant. Even in the studies examined, a multidisciplinary team evaluated the patient before proceeding to surgery [16].

Therefore, we can consider post-radiation fractures as a rare but highly formidable complication, posing challenges for orthopedic surgeons.

To answer the initial question regarding the most promising advances in the treatment of post-radiation fractures, we can certainly affirm that given the complexity of treatment and management of complications, the most appropriate move for the orthopedic surgeon is to prevent such fractures. Currently, the most promising results are provided by prophylactic nailing concerning fracture prevention. 

Once these fractures have occurred, free vascularized fibular flaps appear to have a high success rate in achieving bone union when utilized in revision osteosynthesis during the second or third surgical attempt. Indeed FVG addresses the greatest deficiency of post-radiation fractures: blood supply. Vascularized bone grafts are preferable to the use of iliac crest grafting in the revision of plate osteosynthesis.

As extensively demonstrated, intramedullary nailing represents the gold standard for managing post-radiation fractures in the diaphysis of long bones as a primary treatment. Nailing, furthermore, is a procedure that does not require high surgical skills and allows immediate weight bearing after surgery. In addition, the intramedullary nail has a high cost–benefit ratio, which is favorable for various hospital facilities and healthcare systems.

Megaprostheses are a valid treatment, especially as a primary approach in the metaepiphyseal region, and their success is potentially limited by infection in salvage procedures. Megaprosthesis replacement surgery entails significant costs, including materials, specialist fees, and operating room expenses. It offers substantial benefits in terms of improved quality of life, long-term cost savings, and enhanced functionality for many patients but, given the variability in outcomes, careful patient selection and continuous evaluation of alternative treatments are essential to optimize the cost–benefit ratio of this intervention [21].

The main limitation of this and many other studies on the subject appears to be the restricted number of cases available for study, as well as patient follow up. These are often patients undergoing multiple treatments, leading to unfavorable outcomes due to factors unrelated to the fracture or seeking opinions from various specialists in a sometimes unsuccessful quest for the most suitable treatment. 

It is not always straightforward to compare patients’ medical histories and factors to be considered in the analysis, considering that some patients underwent only one surgery while others underwent three or four. Additionally, some patients experienced fractures immediately after radiotherapy, while others did so years later, resulting in different follow-up durations. Not all studies analyze the same criteria, thus forcing us to consider increasingly stringent parameters

An additional bias is the lack of data on how patients received radiotherapy, with certainty only regarding the total amount of Gy received.

Moreover, information regarding the tumors’ specific locations was unavailable. Studies indicate that tumors situated in the anterior compartment of the thigh exhibit a higher incidence of fractures compared to those in the medial or posterior compartments [22].

## Figures and Tables

**Table 1:** Material and methods.

Autor	Publication	Journal	Title
Tibbo ME, Houdek MT, Bakri K, et al. [12]	November 2020	Journal of Plastic, Reconstructive & Aesthetic Surgery	Outcomes of free vascularized fibular flaps for the treatment of radiation-associated femoral nonunions
Razavian N, et al. [13]	November 2020	International Journal of Radiation Oncology*Biology*Physics	Radiation-Induced Insufficiency Fractures After Pelvic Irradiation for Gynecologic Malignancies: A Systematic Review
Matsuhashi M, et al. [14]	July 2021	Arch Orthop Trauma Surg	Treatment for postoperative infection of pathological femoral fracture after radiotherapy: two case reports and review of the literature
Claxton MR, et al. [15]	April 2020	Journal of Plastic, Reconstructive & Aesthetic Surgery	Utility of free vascularized fibular flaps to treat radiation-associated nonunions in the upper extremity
Sambri A, et al. [16]	August 2021	Arch Orthop Trauma Surg	Femoral fracture in primary soft-tissue sarcoma of the thigh treated with radiation therapy: indications for prophylactic intramedullary nail
Muratori F, et al. [17]	June 2021	Injury	Treatment options in femoral radiation fractures following soft tissue sarcoma: Incidence, risk factors, failures and flowchart of treatment
Lee J, et al. [18]	April 2021	Arch Orthop Trauma Surg	Pathological fractures of the femur after radiation therapy for soft tissue tumor: a case series of seven patients treated with repeated internal fixation
Bretschneider T, et al. [19]	July 2021	International Journal of Surgery Case Reports	Pathologic femur fractures following surgery and radiotherapy for soft tissue sarcomas: A case series

**Table 2:** Articles included in the review.

Author	(mCMS)	Publication	Journal	Title
Sambri A, et al. [16]	49	August 2021	Arch Orthop Trauma Surg	Femoral fracture in primary soft-tissue sarcoma of the thigh treated with radiation therapy: indications for prophylactic intramedullary nail
Muratori F, et al. [17]	61	June 2021	Injury	Treatment options in femoral radiation fractures following soft tissue sarcoma: Incidence, risk factors, failures and flowchart of treatment
Lee J, et al. [18]	30	April 2021	Arch Orthop Trauma Surg	Pathological fractures of the femur after radiation therapy for soft tissue tumor: a case series of seven patients treated with repeated internal fixation
Bretschneider T, et al. [19]	27	July 2021	International Journal of Surgery Case Reports	Pathologic femur fractures following surgery and radiotherapy for soft tissue sarcomas: A case series

**Table 3:** Results, first part.

Series	N. of Patients	N. of Fractured Patients (%)	M/F	PIN	Periosteum Stripping among the Fractured	Age at the PIF **	Fracture Onset Months after RT	Site of Fracture
Muratori F. et al. [17]	570	28(5)	9/19	0	23	60	70	Femur 20, Tibia 4, humerus 2, ulna 1, radio 1
Bretschneider et al. [19]	NA *	3	1/2	0	2	57	108	Femur 3
Jongseok Lee et al. [18]	NA *	7	1/6	0	32	58	50	Femur 7
Sambri et al. [16]	540	15(3)	6/9	11	2	56	52	Femur 15

* Case report with description of individual fractured patients; ** Post-insufficiency Fracture.

**Table 4:** Results, second part.

Series	First Fracture Treatment	Primary Outcome	Further Treatment (Second Treatment)
Muratori F. et al. [17]	Nail 15 Plate fixation 3 Conservative 3 Amputation 3 Cannulated screws 1 Prosthetic replacement 3	Union 9 Union after revision 1 Non-union 6 Infection 3	Ostheosynthesis revision1(plate + bone graft) Amputation 2 Prothesis 5
Bretschneider et al. [19]	Nail 2 Plate fixation 1	Non-union 2 Died (metastasis)	Ostheosynthesis revision (thicker nail) Amputation
Jongseok Lee et al. [18]	Nail 4 Plate fixation 1	Non-union 2 Died (metastasis)	Ostheosynthesis revision 4 (1 plate + bone graft, 1 thicker nail, 1 dynamization, 1 blocking screws insertion)
Sambri et al. [16]	Refused 4 Nail 7 Plate fixation 1 Prosthetic replacement 3	Union 1 Non-union 7	Megaprothesis 4 Ostheosynthesis revision1 (plate + bone graft)

## Data Availability

The datasets used and/or analyzed during the current study are available from the corresponding author on reasonable request.
